# A quantum probabilistic framework for reasoning coherence under contextual variability

**DOI:** 10.3389/fcogn.2026.1727891

**Published:** 2026-02-11

**Authors:** Geoffrey Whittle-Walls

**Affiliations:** 1 The Humanology Lab, Denver, CO, United States; 2 King's College London, London, United Kingdom

**Keywords:** contextual reasoning, decision making, interference effects, quantum cognition, rational-order axioms, social cognition, superposition and entanglement

## Abstract

Human reasoning is traditionally modeled through rational-order frameworks that assume stability, separability, and coherence. Yet across judgment, valuation, perception, and social decision-making, empirical work consistently reveals patterned violations of these assumptions. These deviations intensify in real-world contexts shaped by institutional constraints, identity commitments, and collective narratives, where reasoning must navigate incompatible interpretive frames and interdependent evaluative pressures. Existing theories typically treat such phenomena as noise, bias, or bounded rationality, leaving no formal account of how rational-order rules interact with the variability inherent in social domains. This article proposes a structural framework that explains why these two regimes diverge and how their interaction produces systematic mismatches. Building on quantum probability theory, not as a physical metaphor but as a representational tool, it formalizes evaluative states that remain indeterminate until elicited, transform under contextual modulation, and become relationally coupled across agents and domains. Whereas, existing quantum-cognition models primarily address task-level effects such as conjunction errors or order dependence, this framework scales quantum principles to socially and institutionally embedded reasoning. The account identifies contradiction, interference, entanglement, and resolution as recurrent properties of real-world cognition and shows how quantum formalisms provide a coherent vocabulary for capturing these phenomena. To support cumulative progress, the article outlines a research agenda with empirically testable designs for distinguishing incompatible bases, assessing inseparability, modeling context-driven transformations, and integrating multi-level reasoning environments. This program positions quantum and classical approaches within a unified architecture and advances a broader science of reasoning.

## 1 Introduction

Human reasoning has long been modeled through rational-order frameworks that treat judgment and decision-making as rule-guided processes grounded in stability, separability, and coherence. Expected utility theory and Bayesian inference formalize this view by assuming that preferences remain invariant across contexts, that beliefs update consistently, and that options can be evaluated independently of the environment in which they appear (Von Neumann and Morgenstern, 1947, 2007; Savage, 1954; Sen, 1971). These models offer analytical clarity, but they also prescribe an idealized view of how reasoning ought to function. Consequently, departures from these principles, contextual shifts, preference reversals, order effects, and seemingly incompatible judgments are often interpreted as cognitive noise, bias, or limited optimization (Slovic and Tversky, 1974; Tversky and Kahneman, 1974; Kahneman, 2011).

Yet substantial empirical evidence challenges the idea that such deviations are merely errors. In domains such as judgment, valuation, categorization, and perception, violations of invariance and separability consistently emerge as organized patterns observed across populations and experimental methods (Kahneman and Tversky, 1979; Tversky and Kahneman, 1981; Tversky and Shafir, 1992; Slovic, 1995; Booij et al., 2010). These patterns are notably more noticeable in social and institutional settings where uncertainty, changing norms, relational pressures, and identity commitments are prevalent. In these contexts, reasoning frequently does not follow the stable, context-free structures assumed by rational models (Kuran, 1995; Sunstein, 2001; Guess et al., 2020). What appears inconsistent with classical axioms often reflects locally coherent responses to social constraints, interpretive frames, and institutional expectations (Fiske and Taylor, 1991; Lodge and Taber, 2013; Gidron and Hall, 2017).

Quantum cognition research partly arose to address this gap. Instead of viewing contextual variability as cognitive failure, quantum-probabilistic models formalize how evaluative states can stay indeterminate before being prompted, how judgments are influenced by context, and how interconnected evaluations create non-separable outcome structures (Busemeyer and Bruza, 2012). Empirical evidence shows that violations of conjunctions, order effects, and interference patterns can be explained with quantum concepts like superposition, contextual change, and entanglement (Aerts et al., 2013; Busemeyer et al., 2006; Bruza et al., 2015; Wang et al., 2013, 2014). Recent reviews have unified these findings and highlighted their significance for decision theory and cognitive modeling (Pothos and Busemeyer, 2022).

Despite progress, the reasoning contexts with the greatest real-world impact, those structured by institutions, group identities, informational asymmetries, and collective narratives, are still relatively under-theorized in quantum cognition. In such settings, individuals face competing interpretive frameworks, normative pressures, and relational commitments across multiple levels of social organization. Reasoning here involves more than the formation of preferences or the updating of probabilities; it requires engaging with systems of meaning that both constrain evaluation and enable contextual reconfiguration (Benkler et al., 2018; Cinelli et al., 2021; Jasanoff, 2010; Mason, 2018; Iyengar et al., 2019). This article contends that the structural features of quantum cognition, indeterminacy, interference, non-separability, and measurement-induced stabilization, also apply to reasoning in these broader contexts and can provide a useful framework for understanding how coherence is influenced by context.

This theoretical article makes four principal contributions to the understanding of contextual reasoning by proposing a representational architecture that elucidates how context-dependent evaluation markedly diverges from rational-order assumptions. Firstly, it introduces a dual-regime framework that differentiates rational-order reasoning, characterized by stability, separability, and rule-based coherence, from contextual reasoning, which entails relational dependence, indeterminacy, and the capacity for dynamic reconfiguration. Secondly, it underscores systematic upward and downward incompatibilities between these regimes, illustrating how formal axioms often fail to encompass evaluations influenced by social factors and how contextual reasoning consistently deviates from rational-order assumptions in structured, non-random manners. Thirdly, it presents a representational model grounded in quantum-probabilistic principles, functioning as a formal grammar to articulate these incompatibilities without dependence on specific task dynamics or algorithmic models. Lastly, it broadens the scope of quantum cognition beyond laboratory phenomena by demonstrating the applicability of non-classical structures to identity-related, institutional, and collective reasoning contexts.

This approach builds on existing reviews. Although Pothos and Busemeyer (2022) offer a detailed overview of quantum cognition and Huang et al. (2025) compile empirical results from various paradigms, neither provides an architectural framework connecting quantum principles to the structural conflicts between rational-order models and socially embedded reasoning. The current work addresses this by outlining the representational scaffolding necessary to explain why these tensions emerge and how they can be systematically resolved across different domains.

The article does not assert that quantum theory presently offers a comprehensive model of human reasoning within intricate social contexts. Instead, it delineates the fundamental structural requirements for such integration and elucidates how non-classical representations coordinate the interaction between coherence norms and contextual reasoning. Consequently, the contribution is primarily architectural and integrative in nature. By representing contextual frames as incompatible measurement bases, the framework yields empirical predictions such as persistent order effects, non-additive probability patterns, and identity-conditioned interference, which cannot be derived from classical or Bayesian models without supplementary mixture assumptions or the redefinition of context-specific parameters. These predictions provide a foundation for direct empirical comparison among competing representational paradigms.

## 2 Formal rational-order models of reasoning

### 2.1 Concept and variants

Rational-order reasoning refers to formal systems that model judgment as a rule-governed process defined by stability, coherence, and independence. Expected-utility theory (Von Neumann and Morgenstern, 2007) and Bayesian probability (Savage, 1954) constitute the classical foundations of this approach, providing axioms that render preferences and beliefs mathematically tractable. In cognitive science, Bayesian models extend these principles by treating cognition as probabilistic inference governed by systematic updating of priors and likelihoods (Griffiths et al., 2008).

Stability requires that preferences remain invariant across repeated presentations of equivalent choices and permits their representation by a continuous utility function. Coherence demands adherence to the axioms of probability, including belief updating via Bayes' rule.


$$\begin{array}{l} P\left(H|E\right)\propto P\left(E|H\right)P\left(H\right). \end{array}$$

Independence asserts that irrelevant contextual changes should not overturn established preferences. Formally, if an agent prefers *A* to *B*, that preference must persist when each is mixed proportionally with a common alternative *C* at weight λ, where 0 < λ < 1:


$$\begin{array}{l} A\;≻B\Rightarrow \lambda A\;+\left(1-\lambda \right)C≻\lambda B+\left(1-\lambda \right)C. \end{array}$$

The axiom encodes an invariance requirement: as long as the shared component *C* and the mixing weight λ are held constant, the relative standing of *A* and *B* should not depend on contextual embedding. When taken together, these principles yield a system in which utilities are linearly additive, probabilities are additive, and consistency across contexts is treated as a normative benchmark for rational action.

A broad family of models adapts these principles without abandoning them. Bounded rationality (Simon, 1957) softens assumptions about computational capacity while preserving optimization within local constraints. Procedural rationality (Simon, 1976) reframes decision-making as a process but assumes stable outcomes. Ecological rationality (Gigerenzer and Selten, 2001) interprets coherence as environmental fit rather than logical necessity. Strategic and behavioral game-theoretic models (Myerson, 2007; Camerer, 2011) extend rational choice to interactive domains without relinquishing equilibrium coherence, while fairness-based and resource-rational approaches (Fehr and Schmidt, 1999; Lieder and Griffiths, 2020) reinterpret optimization in moral or computational terms. Across these variants, coherence remains the evaluative standard, and departures such as preference reversals or context effects are framed as noise, bounded optimization, or misalignment rather than as evidence of alternative reasoning logics. The rational-order framework, therefore, functions as a self-stabilizing system that assimilates anomalous findings through parameter adjustment while preserving its foundational axioms.

### 2.2 Empirical evidence

**Figure 1:**
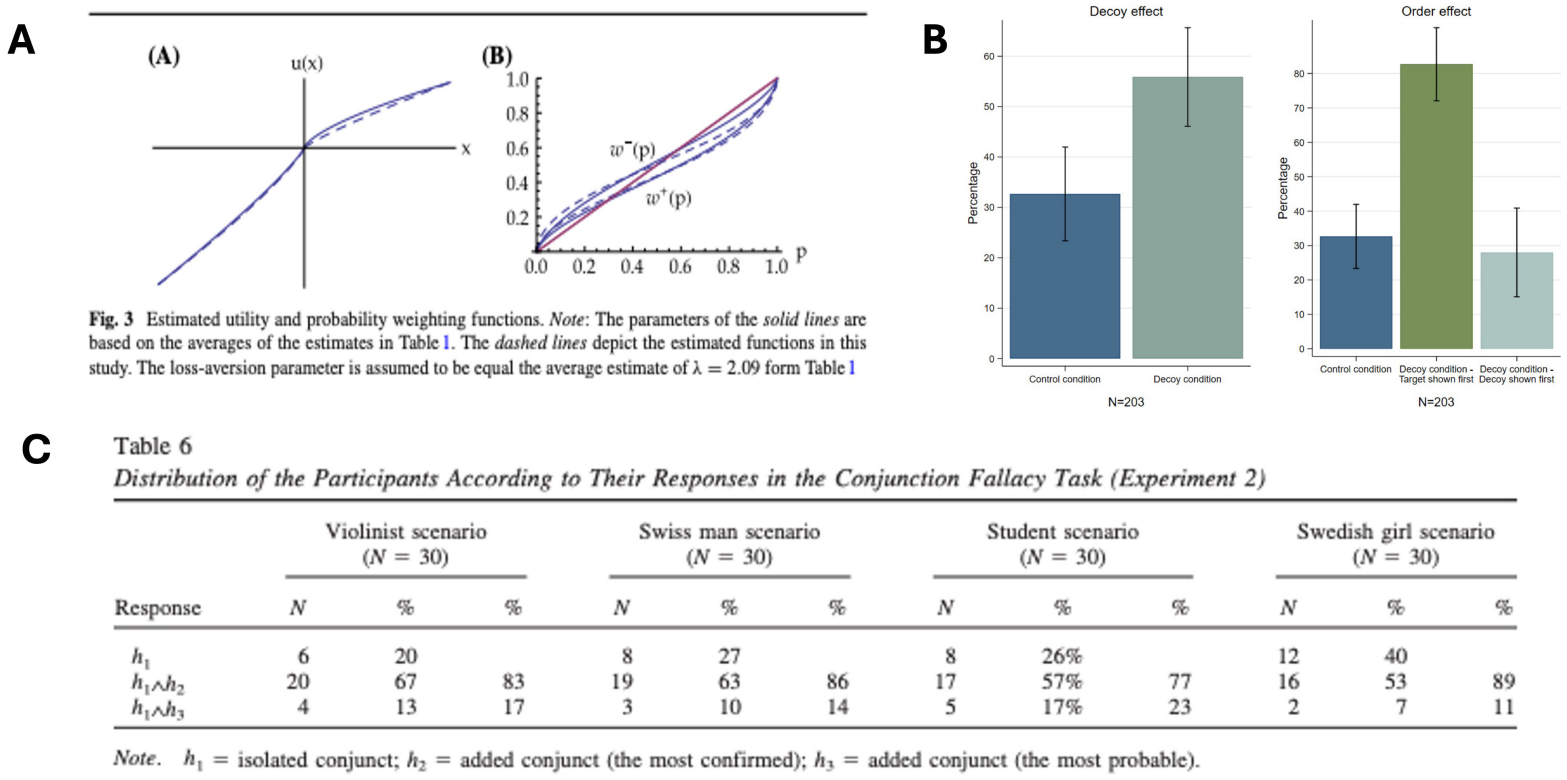
Selected evidence of systematic departures from the rational-order system. **(A)** Estimated utility and probability weighting functions in a representative population, showing concave gains, convex losses, and probability distortions inconsistent with stable preferences (Data: Booij et al., 2010). **(B)** Distribution of responses in conjunction fallacy tasks across multiple scenarios, with a majority selecting the more confirmed but logically inconsistent option (Data: Tentori et al., 2013). **(C)** Field experiment on survey participation showing that the introduction of a decoy alternative increases target choice and that presentation order further amplifies the effect, violating independence of irrelevant alternatives (Data: Stoffel et al., 2023).

A wide empirical literature documents patterned violations of stability, coherence, and independence, as summarized in Figure 1, and these violations appear precisely in contexts where rational-order models predict invariance. In valuation, prospect theory identifies non-linearities in subjective utility and probability weighting: losses carry disproportionate psychological weight relative to gains, and probability weighting follows an inverse-S-shaped curve (Tversky and Kahneman, 1981; Booij et al., 2010). These findings contradict the assumptions of linear integration and stable utility. In judgment, the conjunction fallacy demonstrates that detailed descriptions can be judged more probable than broader categories, a phenomenon that is widely replicated across cultures (Tentori et al., 2013) and is incompatible with the additive structure of classical probability. In choice, phenomena such as asymmetric dominance (Huber et al., 1982) and predictable order effects (Ariely and Norton, 2008; Stoffel et al., 2023) show that adding a normatively irrelevant option or rearranging the sequence of presentation reliability alters preferences, violating separability and independence. These patterns are neither sporadic nor task-specific; they form domain-general regularities that persist even under incentive-compatible conditions.

### 2.3 Causes and processes

Classical explanations account for these regularities while retaining coherence as the normative anchor. Cognitive limitations (Simon, 1957; Newell and Simon, 1972), affective and motivational influences (Damasio, 2004; Bechara et al., 1997), computational bottlenecks (Sims, 2003; Gabaix, 2011; Gershman, 2019; Gabaix et al., 2003), and ecological adaptation (Gigerenzer and Todd, 1999; Henrich, 2016) have all been proposed as mechanisms underlying non-classical behavior. Research on working memory interference (Baddeley and Hitch, 1974) further suggests that variability often reflects structured interference processes rather than decay or error. These accounts enrich the description of how deviations arise but preserve the idea that inconsistency is a deviation from rational form, not an inherent characteristic of reasoning itself. As a result, they do not fully address empirical cases in which contradiction, non-separability, or context dependence appears intrinsic to the phenomenon under study.

The systematic recurrence of these effects exposes a fundamental misalignment between rational-order models and the properties of social domains. Empirical phenomena such as framing and asymmetric dominance demonstrate that the meaning and evaluative weight of an option shift with contextual embedding, violating assumptions of independence and separability. Order effects further reveal that judgments are not retrieved from pre-existing preference states but evolve as information is encountered, contradicting the premise that evaluations are fully specified prior to elicitation. In cases where the evaluation of one element depends inseparably on the presence or interpretation of another, the cognitive system resists decomposing into independent components.

These patterns indicate forms of reasoning in which evaluative states are dynamically constituted, relational dependencies are intrinsic, and coherence is achieved contextually rather than imposed axiomatically. Such properties correspond to contextuality, non-commutativity, and entanglement-like dependencies that cannot be represented by additive probability measures or separable utility functions. More formally, when evaluative frames correspond to non-commuting informational partitions, no single joint probability distribution can satisfy the axioms of classical coherence across both frames simultaneously. Preserving classical consistency under these conditions requires suppressing one frame, averaging across contexts, or redefining the state space *post hoc*. These strategies maintain formal closure while sacrificing representational fidelity, indicating the need for a framework in which incompatibility is modeled as a structural property of reasoning rather than as error, noise, or bounded rationality.

## 3 Contextual reasoning in social domains

Reasoning in social domains unfolds within environments that are themselves structured systems. These environments are composed not only of other individuals or groups, but of institutional rules, shared narratives, symbolic boundaries, communication infrastructures, and incentive regimes that jointly shape what can be noticed, articulated, justified, or acted upon (Mercier and Sperber, 2017; Huang et al., 2026). Contextual reasoning, therefore, cannot be understood as the application of stable internal preferences to external information. It is the process through which evaluative orientations are formed, constrained, and expressed within a dynamically organized social space. A defining feature of such domains is that evaluative meaning is not intrinsic to information. The same factual claim, behavioral option, or policy proposal can acquire different significance depending on its placement within institutional categories, identity narratives, or normative expectations. Research across social psychology, political science, and organizational sociology shows that credibility, relevance, and moral valence are assigned through social interpretation rather than inferred directly from content (Fiske and Taylor, 1991; Fiske, 2000; Douglas and Wildavsky, 1982; Kahan, 2017; Gidron and Hall, 2017; Vosoughi et al., 2018). As a result, reasoning in social contexts is inherently contextual: evaluations depend on how information is framed, who communicates it, which norms are salient, and what consequences attach to endorsement or rejection.

These environments exert influence through structural constraints, not merely through persuasion or incentives. Institutional arrangements define categories of legitimacy and authority; social norms regulate expressive boundaries; communication systems amplify some interpretations while suppressing others (van der Linden et al., 2020); and narrative frameworks link discrete judgments into broader stories of threat, belonging, or obligation (March and Olsen, 1989; Sunstein, 2001). Together, these elements shape a field of admissible evaluations within which reasoning occurs. Individuals do not select freely from this field; they navigate it under conditions of visibility, sanction, coordination, and risk.

Importantly, social environments operate as feedback systems. Expressions of judgment alter the environment that subsequent judgments respond to. Public alignment with dominant interpretations reinforces their apparent coherence and stability, while strategic silence, hedging, or coded dissent can sustain latent disagreement without overt conflict. Over time, these feedback processes can stabilize collective orientations that obscure underlying diversity, or under conditions of disruption, produce abrupt reconfigurations when previously constrained interpretations become expressible (Kuran, 1995; Sunstein, 2001; Centola et al., 2018). Reasoning thus both responds to and reshapes the social environment in which it is embedded.

In such contexts, individuals consistently maintain multiple evaluative orientations that respond to varying contextual requirements. An individual may privately support one interpretation while publicly demonstrating another; may employ different reasoning across institutional environments; or may alter their evaluative stance as the audience, framing, or perceived sanctions change. These variations are not solely attributable to preference instability or strategic manipulation. Instead, they reflect the activation of distinct contextual constraints, each producing locally coherent reasoning within its respective domain.

At the collective level, these dynamics produce patterns that cannot be solely explained by the aggregation of individual beliefs. Groups frequently exhibit coordinated shifts in judgment in response to changes in symbolic framing, leadership cues, or institutional cues, even when individual attitudes remain diverse. Such coordination emerges because social environments shape interpretive processes based on shared meanings rather than shared preferences. Consequently, collective reasoning reflects the organization of the interpretive space itself, rather than the convergence of internal belief systems.

These dynamics motivate four core constructs that define how people reason in social contexts. Identity serves as a socially grounded frame that shapes perception and influences evaluative judgments, highlighting which interpretations are considered critical, believable, or threatening in a given context (Tajfel and Turner, 2004; Mason, 2018). Social interference occurs when multiple identity-related or norm-driven evaluative attitudes are present simultaneously, but cannot all be satisfied, resulting in a structured tension rather than outright contradiction (Kunda, 1990; Smith and DeCoster, 2000). Social entanglement refers to the inseparable connections among evaluative elements across judgments, identities, or institutional roles, such that the evaluation of one element depends on others (Fiske A. P., 1992; Gidron and Hall, 2017). Coherence, in this context, doesn't mean universal consistency across all possible perspectives but rather context-dependent alignment: reasoning is coherent when it meets the interpretive, normative, and relational standards of the environment where it occurs (Festinger, 1957; Druckman and Levendusky, 2019).

Each construct admits observable behavioral signatures that allow these distinctions to be operationalized. Identity manifests as systematic shifts in the order of judgment, salience, or weighting across contexts. Coherence appears as locally stable response patterns that cannot be jointly maintained across incompatible frames. Social interference is observed as non-additive changes in response probabilities when multiple contexts are active. Social entanglement is reflected in coordinated shifts across judgments, agents, or institutional positions that cannot be decomposed into independent effects without loss of explanatory power.

These constructs do not describe independent mechanisms. They characterize complementary facets of a single contextual reasoning architecture in which identity commitments, interpretive frames, and relational positioning jointly structure the evaluative space within which judgments are expressed. Variability in reasoning, therefore, does not signal a breakdown of rational organization. It reflects the fact that coherence is achieved relative to contextually constrained representational spaces rather than imposed axiomatically across environments.

Within social domains, coherence must therefore be defined as context-relative alignment rather than global consistency. A judgment is coherent when it satisfies the interpretive, normative, and relational constraints imposed by a given social environment. These constraints specify which evaluative orientations are admissible, which combinations are prohibited, and which transitions between orientations are socially intelligible. Apparent inconsistencies across contexts reflect movement among environments governed by different constraint structures, rather than instability of preference (March and Olsen, 1989; Druckman and Levendusky, 2019).

Reasoning in such environments consequently exhibits three structural properties. First, multiple evaluative orientations may coexist because distinct social frames impose incompatible but locally valid constraints. Second, dependencies among judgments arise because identity positions, institutional roles, and normative expectations couple evaluative dimensions that cannot be independently specified. Third, resolution occurs only when a specific context enforces selection among competing orientations, stabilizing expression without eliminating latent alternatives. These properties pertain to constraints on representational organization rather than to psychological mechanisms. As such, they impose representational requirements that exceed those typically assumed in classical probabilistic models of judgment, motivating a formal analysis of representational adequacy (Busemeyer and Bruza, 2012; Pothos and Busemeyer, 2022).

## 4 Discussion

### 4.1 Systematic incompatibilities between contextual and formal models of reasoning

**Figure 2:**
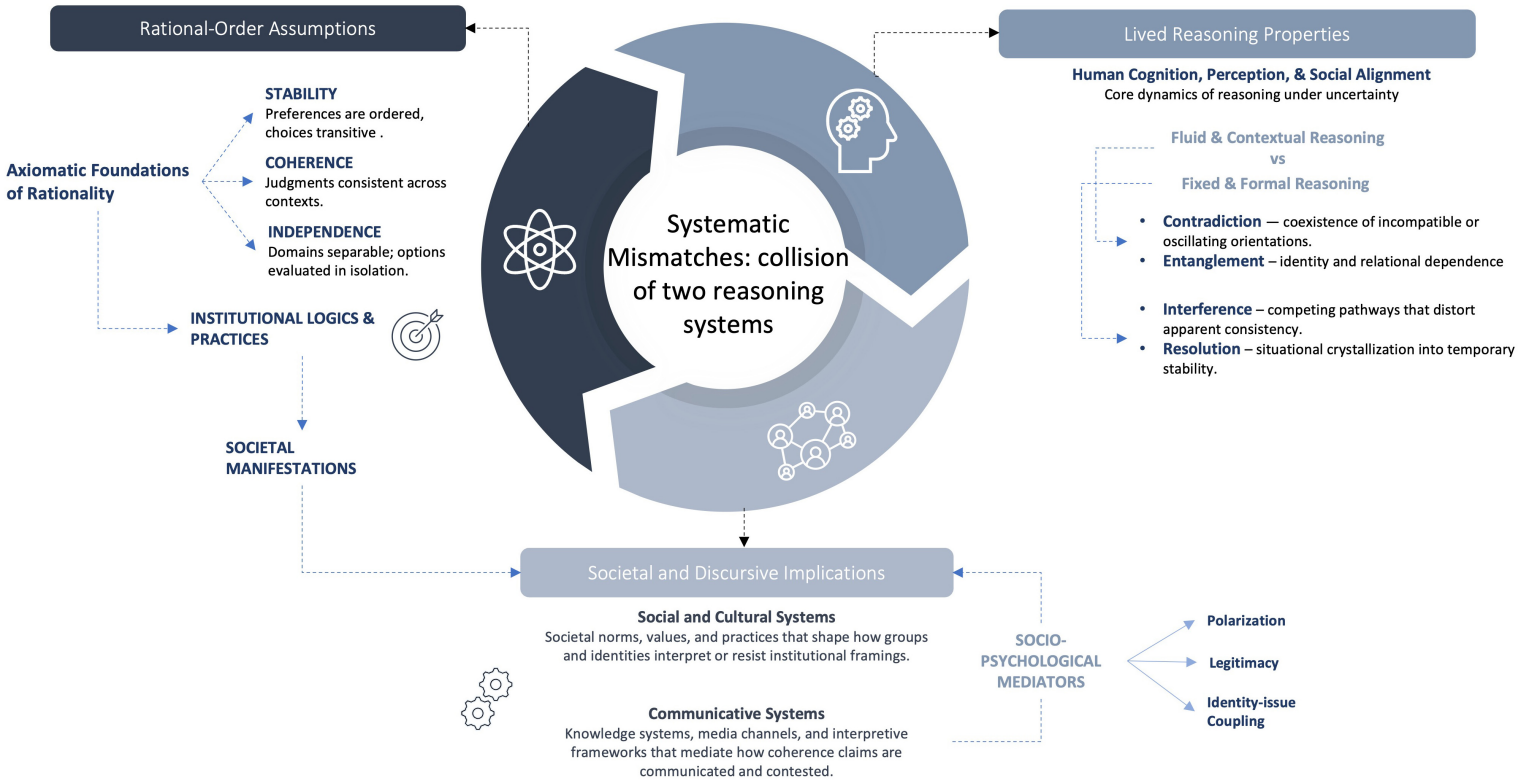
Conceptual model of systemic interference between formal and contextual reasoning. Arrows depict the bidirectional dynamics through which formal coherence exerts downward constraint on reasoning environments while contextual reasoning generates upward adaptation that reconfigures formal frameworks. Together, these interactions define the recursive feedback system examined in Systematic Incompatibilities between Contextual and Formal Models of Reasoning.

Rational-order reasoning and contextual reasoning are founded on fundamentally distinct logics. While empirical research in judgment and decision-making increasingly suggests that deviations from classical axioms are not arbitrary but rather reflect a structural tension between these two reasoning types. Rational models enforce coherence through axiomatic constraints, presuming that preferences are stable, separable, and consistent across various contexts. Conversely, reasoning in real-world social environments depends on context-dependent constraints, in which evaluative distinctions are conditionally activated, interdependent, and weighted differently by institutional, relational, and situational factors (see Figure 2). Institutionalized models, such as policy rules, metrics, or algorithms, impose top-down constraints that guide the perception of acceptable or rewarded reasoning. Nonetheless, when individuals perceive these constraints as misaligned with their lived experiences, they often reinterpret their meanings, shifting coherence into frameworks of identity, morality, or symbolism. These disruptions are organized and directional rather than accidental, representing opposing forces that influence the stabilization and understanding of reasoning.

#### 4.1.1 Downward impacts: how coherence ideals reshape reasoning environments

Downward impacts refer to the way coherence principles, once embedded in formal models, actively restructure the environments in which reasoning occurs. When institutionalized through administrative rules, evaluative criteria, performance metrics, algorithmic scoring systems, or policy design, coherence assumptions cease to function merely as analytical abstractions. Instead, they become operative constraints that define what counts as a valid preference, a legitimate belief, or a rational choice (Callon, 1998; MacKenzie, 2006, 2011). Stability, transitivity, and independence are no longer descriptive ideals but regulatory benchmarks against which actors are evaluated.

This organizational embedding consolidates diverse reasoning processes into a single representational system. As Simon (1957) noted, bounded rationality results not only from cognitive constraints but also from the simplified structures that render behavior understandable within organizational frameworks, a concept further expanded by Gigerenzer and Todd (1999). In such systems, variability is not removed but shifted. Incentives for coherence encourage actors to demonstrate stability where measurement occurs, while unresolved ambiguity is pushed into unmeasured, insulated, or strategically controlled areas. This process explains Goodhart's Law: when coherence is targeted, it loses its value as an indicator (Goodhart, 1984; Strathern, 1997).

Empirical studies of audit cultures demonstrate how evaluative feedback induces performative stability even when underlying processes remain volatile (Espeland and Sauder, 2007). Apparent consistency is maintained through continual micro-adjustment beneath the surface, producing smooth aggregate indicators that mask clustered volatility, an effect analogous to volatility clustering in financial systems (Mandelbrot, 1963; Lux, 1998; Gabaix et al., 2003). In this way, coherence ideals do not merely describe reasoning; they actively shape it, generating environments that appear orderly while remaining internally non-stationary.

At this level, the analogy to quantum “collapse” becomes conceptually sound, though strictly structural rather than physical. Institutional measurement selectively stabilizes one evaluative frame, suppressing alternative interpretations that remain latent yet behaviorally consequential. Measurement fixes a provisional representational state, rendering it actionable and legible, while background variability persists outside the formal account. Rationality thus shifts from a cognitive property to a control technology: a formal apparatus that enforces representational order and then interprets the discrepancies it produces as deviations.

#### 4.1.2 Upward impacts: how contextual reasoning reorganizes formal structures

The reciprocal dynamic operates through upward impacts, which trace how contextual reasoning adapts to, reinterprets, and ultimately reshapes the formal systems that constrain it. When coherence norms restrict the admissible expression of uncertainty, contextual cognition compensates by relocating meaning into alternative evaluative domains. Under such conditions, identity emerges as a substitute coherence principle, anchoring judgment in group-defined norms of relevance, credibility, and value, thereby providing stability when formal criteria are inaccessible, insufficient, or misaligned with lived experience. Rather than eliminating uncertainty, identity-based reasoning reorganizes it by filtering evidence through group-congruent evaluative standards, restoring predictability (Kahan, 2013; Mason, 2018).

Across governance, communication, and policy settings, intensified coherence constraints often displace interpretive authority into moral, affective, or symbolic registers. Empirical work shows that as formal systems tighten their representational demands, evaluative dimensions such as fairness, legitimacy, threat, and belonging become structurally coupled with instrumental assessments (Fiske S. T., 1992; Jasanoff, 2010; Latour, 2007). These couplings generate dependencies that cannot be decomposed into independent attributes. From a modeling perspective, they resemble entanglement-like relations: changes in one evaluative dimension reconfigure the contextual basis of others, producing interference effects without implying internal inconsistency.

A central consequence of these upward dynamics is the fragmentation of reference frames. Disagreements arise not only over factual claims, but over what qualifies as evidence, which authorities are credible, and which risks are actionable. Coherence becomes locally internally stable within groups yet decoherent across them. Multiple, internally consistent but mutually incommensurable evaluative frameworks coexist without a shared calibration. This pluralization of epistemic fields undermines coordination based on shared axioms, forcing governance, and collective decision-making to rely instead on negotiated alignment and credibility (Zhang and Busemeyer, 2021).

Taken together, downward and upward impacts form a recursive feedback system in which reasoning environments and cognitive processes mutually constrain one another. Coherence idealizations stabilize evaluative representations by privileging low-variance structures aligned with institutional norms, while contextual variability reorganizes these structures by shifting which distinctions are salient and jointly maintainable. Stability, coherence, and independence, therefore, appear not as universal properties of reasoning but as contingent outcomes sustained through ongoing environmental regulation and adaptive response.

When stabilization and reconfiguration operate simultaneously, classical probabilistic representations lose adequacy. The resulting patterns are not random deviations but structured consequences of incompatible representational constraints acting across levels. Downward impacts enforce particular evaluative bases; upward impacts induce effective basis transformations through repeated contextual engagement. The tension that emerges reflects a principled incompatibility between reasoning regimes rather than error or noise, motivating the need for a formalism capable of representing contextual dependence, non-separability, and stabilization under incompatibility.

### 4.2 Quantum theory as a representational solution

To demonstrate quantum cognition as a representational approach, belief formation in highly salient social domains is modeled as the contextual stabilization of an evaluative state, rather than as the retrieval of a context-independent preference. The main assumption is that evaluative dimensions are often incompatible, meaning they cannot be jointly maintained within a single basis, and that the context determines which distinctions can be coherently stabilized during judgment. This representational logic extends beyond individual cognition, applying equally to social-reasoning environments, networks of individuals, institutions, and situations in which shared constraints, rather than isolated mental states, shape coherence as an intrinsic property of reasoning.

#### 4.2.1 State space and evaluative degree of freedom

Formalizing belief stabilization within a quantum cognition framework requires first specifying the representational space in which competing evaluative orientations are jointly encoded. Let *H* be a finite-dimensional Hilbert space representing the respondent's evaluative state over a set of competing orientations. For generality across domains, we decompose the space into two coupled factors:


$$\begin{array}{l} H=H_{E}⊗H_{I}, \end{array}$$

where *H*_*E*_ encodes issue-evaluative content (e.g., threat vs. safety, benefit vs. harm, trust vs. distrust) and *H*_*I*_ encodes identity-and affiliation-relevant structure (e.g., institutional trust alignment, group-based interpretive commitments). The joint cognitive state is represented as a density operator.


$$\begin{array}{l} \rho \;\in D\left(H\right),\;p≽0,\;Tr\left(\rho \right)=\;1. \end{array}$$

Using a density operator rather than a pure state is not merely a technical convenience: it captures empirically common conditions in the target domains, heterogeneous latent orientations, unresolved ambivalence, and partial stabilization under repeated exposure, without collapsing these into *ad hoc* “noise” terms.

#### 4.2.2 Context as an institutional transformation (beyond unitary rotation)

Having specified the structure of the evaluative state, the next requirement is a formal account of how contextual conditions act on that state prior to judgment. From here, let *c* index a context condition (e.g., breaking-news frame, authority statement, social cue, platform-mediated information regime, institutional directive, salient identity prime). Here, I represent context as a completely positive trace-preserving (CPTP) map.


$$\begin{array}{l} E_{c\;}:D\left(H\right)\to D\left(H\right),\;\rho \;\mapsto {\rho \;}_{c}\\ =\underset{k}{\sum }K_{c,k}\rho \;K_{c,k}^{†},\;\underset{k}{\sum }K_{c,k}^{†}K_{c,k}=I. \end{array}$$

This formulation subsumes the unitary transformations commonly used in quantum cognition (when $E_{c}\left(\rho \right)=U_{c}\rho U_{c}^{†}$) but is strictly more expressive and better aligned with institutional environments. In particular, non-unitary components of *E*_*c*_ naturally represent selective stabilization (decoherence-like narrowing of admissible evaluative distinctions) induced by institutional messaging, platform filtering, or normative constraint precisely the “downward” pressures described earlier. Operationally, context is manipulated, not fitted: *c* corresponds to experimental conditions (message frames, order, authority cues) or naturally occurring regimes (pre/post-event windows, institutional announcements).

#### 4.2.3 Measurement as response stabilization (POVMs)

Because contextual transformation alone does not yield observable beliefs, a distinct representational step is required to model how stabilized judgments are elicited as responses. Here, observed judgments are modeled as measurements on the context-transformed state ρ_*c*_. Let y∈Y denote a discrete response category (e.g., endorse/reject, support/oppose, high/low trust). The probability of response *y* under context *c* is given by a positive-operator-valued measure (POVM) ${\left\{M_{y}^{\left(Q\right)}\right\}}_{y\in Y}$, where:


$$\begin{array}{l} M_{y}^{\left(Q\right)}≽0,\;\underset{y\in Y}{\sum }M_{y}^{\left(Q\right)}=I\; \end{array}$$

and


$$\begin{array}{l} \mathrm{Pr}\left(Y=y|c\right)=Tr\left(M_{y}^{\left(Q\right)}\rho_{c}\right)=Tr\left(M_{y}^{\left(Q\right)}E_{c}\left(\rho \right)\right). \end{array}$$

The superscript (*Q*) emphasizes that the measurement operator is question or task-specific, not universal: different survey items, elicitation formats, or institutional response categories correspond to different POVMs. This is critical in the target domains because the “same belief” (e.g., threat perception) can be operationalized through different prompts whose measurement operators need not commute.

#### 4.2.4 Sequential elicitation and non-commutativity

When multiple evaluative judgments are elicited, the order in which measurements occur becomes representationally consequential rather than procedurally incidental. A wide range of empirically observed deviations from classical reasoning, including belief reversal under shifting contexts, polarization following shared evidence, and order-amplified choice effects, are expressed through systematic order sensitivity. To capture this systematically within a quantum cognition framework, let *Q*_1_ and *Q*_2_ denote two elicited judgments (e.g., trust in authority, then perceived threat; benefits, then risks). Represent each judgment as a POVM with elements $\left\{M_{a}^{(Q_{1)}}\right\}$ and $\left\{M_{b}^{(Q_{2)}}\right\}$. In sequential elicitation, the joint response probability depends on measurement order:


$$\begin{array}{l} \mathrm{Pr}\left(a,b|Q_{1}\to Q_{2},c\right)=Tr(M_{b}^{\left(Q_{2}\right)}\;E_{c}\left(M_{a}^{\left(Q_{1}\right)}\left(\rho \right)\right), \end{array}$$

where $M_{a}^{\left(Q_{1}\right)}$ denotes the state-update map associated with outcome *a*. In the projective special case $M_{a}^{\left(Q_{1}\right)}\left(\rho \right)=\frac{\Pi_{a}^{\left(Q_{1}\right)}\rho \Pi_{a}^{\left(Q_{1}\right)}}{Tr\left(\Pi_{a}^{\left(Q_{1}\right)}\rho \right)}$, but the POVM formulation allows graded or coarse response categories typical of surveys. A defining prediction of representational incompatibility is that reversing the order yields different response distributions:


$$\begin{array}{l} \mathrm{Pr}\left(\cdot |Q_{1}\to Q_{2},\;c\right)\neq Pr\left(\cdot |Q_{2}\to Q_{1},c\right), \end{array}$$

which corresponds to non-commutativity at the measurement level (i.e., the effective measurement instruments do not commute). This provides a principled basis for modeling order effects as structural, rather than as noise, attention, or memory artifacts.

This representational architecture separates empirical levers in a principled manner. Contextual conditions, such as framing, authority cues, elicitation order, or pre/post-event windows, are treated as manipulated or observed inputs indexed by *c*, which determines *E*_*c*_ up to a chosen parametrization. Quantities estimated from data include the baseline state ρ and, depending on study design, a parsimonious parameterization of *E*_*c*_ and/or the measurement operators $M_{y}^{\left(Q\right)}$. The model further yields structural predictions, including order dependence, violations of classical additivity in sequential judgments, and context-induced shifts that cannot be reproduced by convex mixtures of context-free states without auxiliary assumptions.

### 4.3 Anchor instantiation: crisis-driven belief reversal

Crisis belief reversal constitutes a demanding instantiation of the representational architecture because, within a single empirical setting, expressed beliefs reorganize rapidly, exhibit systematic sensitivity to elicitation order, and diverge sharply under nominally equivalent informational content delivered through different institutional contexts. This conjunction of empirical features engages all components of the representational architecture introduced above: evaluative states represented by a density operator ρ, contextual transformation implemented through *E*_*c*_, and belief reports realized as measurements $\left\{M_{y}^{\left(Q\right)}\right\}$. Applying the architecture to this domain does not involve modifying these primitives; rather, belief reversal is treated as a structural consequence of contextual transformation acting on mixed evaluative states and their subsequent stabilization through non-commuting measurements. Accordingly, the evaluative configuration of an individual at a time *t* is represented as a density operator.

$$\begin{array}{l} \rho_{t}\in D\left(H_{E}⊗H_{I}\right), \end{array}$$

where *H*_*E*_ encodes issue-relevant evaluations (e.g., threat vs. safety, necessity vs. overreaction) and *H*_*I*_ encodes identity- and institution-related orientations (e.g., trust vs. distrust in authorities, alignment vs. alienation). Prior to a crisis trigger, ρ_*t*_ is typically mixed, reflecting unresolved ambivalence, partial commitments, and coexisting evaluative orientations.

A crisis event or authoritative intervention defines a context condition *c* (e.g., emergency declaration, high-salience media coverage, institutional directive). In this domain, crisis events and authoritative interventions instantiate the contextual transformations formalized above, reshaping the evaluative state prior to elicitation rather than adding evidence in the narrow sense.


$$\begin{array}{l} \rho_{t+1}=E_{c}\left(\rho_{c}\right). \end{array}$$

#### 4.3.1 Measurement order and belief stabilization

Non-unitary components of *E*_*c*_ capture selective stabilization effects commonly observed during crises, such as narrowing of acceptable interpretations, amplification of institutionally sanctioned frames, or suppression of alternative evaluative distinctions. Importantly, this transformation operates prior to any explicit elicitation of belief.

Observed belief reports correspond to measurements on the transformed state ρ_*t*+1_. Let *Q*_*T*_ denote a question probing perceived threat (e.g., “How serious is the situation?”) and *Q*_*I*_ a question probing institutional trust (e.g., “How much do you trust official guidance?”). These questions are represented by POVMs $\left\{M_{y}^{\left(Q_{T}\right)}\right\}\;$and $\left\{M_{z}^{\left(Q_{I}\right)}\right\}$, respectively.

During crises, institutional communication and media exposure often implicitly fix the order in which trust and threat judgments are elicited. In the present model, this is represented by non-commuting measurement instruments. Sequential elicitation yields different stabilized states and response probabilities:

$$\begin{array}{l} \mathrm{Pr}\left(y,z|Q_{T}\to Q_{I},c\right)=Tr\left(M_{z}^{\left(Q_{I}\right)}M_{y}^{\left(Q_{T}\right)}\left({\;\rho }_{t+1}\right)\right),\\ \mathrm{Pr}\left(y,z|Q_{I}\to Q_{T},c\right)=Tr\left(M_{y}^{\left(Q_{T}\right)}M_{z}^{\left(Q_{I}\right)}\left({\;\rho }_{t+1}\right)\right), \end{array}$$

where $M^{\left(Q\right)}$ denotes the state-update map associated with measurement *Q*. When the effective instruments associated with *Q*_*T*_ and *Q*_*I*_ do not commute, these distributions differ, producing order effects that are systematic rather than stochastic.

From a representational standpoint, belief reversal occurs when early measurements often implicitly embedded in media exposure or institutional messaging stabilize one evaluative basis at the expense of others. Subsequent elicitation then samples from a state that has already been partially collapsed, yielding sharp shifts in expressed belief even in the absence of new factual input.

The crisis belief reversal case serves as an anchor instantiation because it exercises all components of the representational architecture mixed evaluative states, context-driven transformation, and non-commuting measurements within a single, empirically recognizable phenomenon.

### 4.4 Adjudication criteria and explanatory scope

The contribution advanced here lies in specifying a representational level that is not explicitly formalized in either dominant classical models of judgment or in much of the existing quantum cognition literature. The novelty lies not in the use of quantum formalism but in isolating representational structure from task-specific dynamics, thereby enabling empirical adjudication across domains within a shared formal space.

In classical models of belief and judgment, contextual variability is typically accommodated by varying parameters. Regression-based, hierarchical Bayesian, and signal-detection approaches represent context by shifting utilities, priors, thresholds, or noise parameters while leaving the underlying representational space fixed (McFadden, 2001; Gelman et al., 2013). When identical information yields divergent response distributions across contexts, such models require context-indexed priors or discontinuous parameter updates, thereby fragmenting the probability space. By contrast, the present architecture treats context as a transformation acting on a shared evaluative state prior to elicitation. This representational commitment yields discriminating empirical consequences. Contextual variability is constrained to manifest as structured violations of classical probability, such as non-additivity, order dependence, and joint-judgment dependence that cannot be reproduced by convex mixtures of stable preference types without additional assumptions (Busemeyer and Bruza, 2012; Pothos and Busemeyer, 2022). These signatures provide criteria for adjudicating whether observed variability reflects parameter drift within a classical space or transformation of the representational state itself.

Existing quantum cognitive models address several of these phenomena, but they typically do so at the level of task-specific dynamics. Quantum-walk and sequential update models are designed to reproduce particular experimental effects such as conjunction errors, preference reversals, or response-time patterns within tightly specified paradigms (Busemeyer et al., 2006; Trueblood and Busemeyer, 2011; Yearsley and Jerome, 2016). While these models offer fine-grained accounts of local decision dynamics, their representational assumptions are often bound to specific task structures, limiting comparability across domains. The present framework departs from this approach by formalizing evaluative states, contextual influence, and elicitation independently of any particular task. Contextual effects are represented as transformations within a common state space rather than as task-indexed dynamics. This separation allows empirical findings drawn from distinct paradigms, survey responses, framing experiments, joint judgment tasks, and crisis-driven belief shifts to be compared at the level of representational structure rather than at the level of fitted dynamics.

A further point of differentiation concerns the treatment of uncertainty and ambivalence. Many quantum cognitive models employ pure states and unitary evolution, implicitly modeling agents as occupying well-defined belief states updated coherently through information exposure (Busemeyer and Bruza, 2012). The present architecture instead treats mixed states as the default representation, reflecting empirical evidence that judgments in high-salience social domains often involve persistent ambivalence and partial coherence (Slovic, 1995; Thompson et al., 1995). Non-unitary contextual transformations then capture selective stabilization induced by institutional, social, or identity-based constraints, shifting the explanatory focus from belief updating to belief stabilization under context.

These representational commitments define the explanatory scope of the approach. It targets cases in which contextual variability exhibits discriminating structural signatures: order effects that persist under repetition, framing effects that exceed mixture bounds, and joint judgments that violate marginal selectivity. The framework does not aim to replace task-specific quantum models of decision dynamics, nor does it claim that all contextual effects are quantum in nature. Its contribution lies in providing a shared representational substrate at which empirical findings from different tasks and domains can be evaluated, compared, and accumulated within a coherent formal space.

## 5 Future directions: a research agenda for a cumulative science of reasoning

### 5.1 Key takeaways and research questions

This paper supports four tightly linked conclusions that together motivate a cumulative research agenda. First, deviations from rational-order models, such as order sensitivity, non-additivity, joint dependence, and context-conditioned divergence, characterize a recurring class of empirical phenomena that arise in high-salience domains such as crisis belief, climate-related attitudes, AI trust, and nuclear risk perception. Second, these deviations exhibit a common structural profile, indicating that they arise from shared representational constraints. Third, although these phenomena are widely documented across psychology, political science, sociology, and decision research, they are typically modeled in isolation, either as task-specific effects or as context-induced parameter shifts. Fourth, this fragmentation has limited cumulative inference: existing work richly describes contextual variability but rarely specifies the conditions under which such variability reflects structural properties of evaluative representation.

These conclusions imply that progress depends on a reorientation of empirical inquiry. Rather than asking whether context matters, the central research problem becomes identifying when contextual variability exhibits the discriminating signatures implied by representational incompatibility. This reorientation yields a small set of necessary research questions. Under what conditions do evaluative distinctions fail to coexist within a single representational basis, such that their joint elicitation produces order-dependent stabilization? When do dependencies between judgments exceed what can be explained by correlated priors, shared information, or network structure, indicating non-separable evaluative states rather than classical dependence? How do belief trajectories evolve when order and overlap are constitutive features of judgment, so that contextual transformation precedes elicitation rather than accumulating incrementally through evidence? And how do institutional and social environments selectively stabilize particular evaluative distinctions, producing apparent consensus or polarization without convergence of underlying preferences? Addressing these questions requires designs that treat order, joint elicitation, and institutional framing as diagnostic conditions rather than as procedural complications.

A defining feature of this agenda is that it cuts across disciplinary boundaries while remaining methodologically grounded. The empirical materials required to test these claims already exist in multiple fields but are rarely analyzed within a shared representational framework. In psychology, order effects and preference reversals are often treated as measurement artifacts; in political science, framing and identity-based polarization are commonly modeled through shifting priors or utilities; in sociology, institutional categories are known to stabilize evaluation but are seldom formalized as measurement contexts. Earlier theoretical accounts anticipated this convergence. Matte Blanco's bi-logic framework, for example, distinguished between asymmetric modes of reasoning that preserve differentiation and symmetric modes characterized by associative coexistence (Matte Blanco, 1975, 1988). The present representational architecture provides a formal language for expressing how such modes interact under contextual constraint, enabling evidence across disciplines to be evaluated against shared structural criteria rather than discipline-specific assumptions.

The central challenge for this research program is adjudicatory rather than descriptive. Multiple mechanisms, such as memory processes, conversational pragmatics, attentional dynamics, and social influence, can reproduce surface-level contextual effects. Cumulative progress, therefore, depends on designs that target discriminating constraints, such as persistent violations of total probability and non-additivity exceeding mixture bounds, rather than on overall model fit. Identifiability is critical: representational claims are testable only when experimental manipulations clearly separate what is controlled (contextual conditions, ordering, elicitation format) from what is estimated (state structure, transformation strength, measurement sensitivity).

The explanatory leverage of the framework is strongest in domains characterized by incompatible evaluative dimensions, strong institutional or identity mediation, and high contextual salience. Establishing when these conditions hold and when alternative classical or task-specific quantum models suffice is the necessary next step toward a cumulative science of contextual reasoning.

## 6 Conclusion

This paper has provided a structural account of why mismatches between rational-order models and observed patterns of human reasoning persist across domains. Rather than treating such mismatches as anomalies or cognitive errors, the analysis shows that they reflect a principled incompatibility between coherence-based representational assumptions and the context-sensitive organization of evaluative reasoning. Classical models presuppose the stability, separability, and joint definability of evaluative dimensions; contextual reasoning, by contrast, unfolds through conditional activation, relational dependence, and adaptive stabilization. When these regimes are forced into alignment, systematic distortions emerge not as failures of reasoning, but as signatures of representational mismatch.

By articulating the downward and upward dynamics through which coherence ideals shape reasoning environments and contextual cognition, the framework reframes rationality itself as an emergent and regulated property rather than an intrinsic cognitive invariant. Coherence appears not as a universal baseline, but as a contingent outcome sustained through institutional measurement and adaptive response. This recursive interaction explains why stability can be observed at the surface level while volatility persists beneath the surface, and why identity, affect, and symbolic meaning increasingly serve as organizing principles when formal criteria fail to secure intelligibility.

Within this context, quantum probability theory has been introduced not as a claim about the physical implementation of cognition, but as a representational architecture capable of expressing indeterminacy, contextual transformation, and non-separable dependence as intrinsic features of evaluative states. Superposition and entanglement function here as structural concepts that render contextual variability formally expressible rather than residual. The value of this framework lies not in replacing classical models wholesale, but in clarifying the conditions under which classical assumptions cease to be adequate and specifying alternative representational commitments that can be empirically adjudicated.

Importantly, the contribution of this work is primarily architectural rather than algorithmic. It does not introduce a novel dynamic process model of decision-making that encompasses the full complexity inherent in socially embedded reasoning. Instead, it delineates a representational landscape within which various modeling approaches, classical, quantum, and hybrid, can be systematically compared. By identifying structural signatures such as incompatibility between evaluative bases, order dependence, interference, inseparability, and context-dependent stabilization, the framework redirects the discourse away from philosophical debates concerning rationality and toward empirically testable assertions regarding the efficacy of different representations.

This repositioning has important implications for how reasoning is studied across psychology, political science, sociology, and related fields. A range of phenomena currently interpreted as bias, polarization, or inconsistency may instead reflect lawful responses to incompatible evaluative demands operating across institutional, social, and affective contexts. Treating these effects as representational consequences rather than cognitive defects opens new avenues for cumulative explanation and model comparison, without abandoning normative rigor or empirical accountability.

The broader implication is that coherence, contradiction, and adaptation are not opposing features of reasoning but coexisting aspects of a system operating under contextual constraints. Reasoning stabilizes not by eliminating variability but by continually negotiating it. The framework developed here provides a formal vocabulary for expressing that negotiation and a foundation for assessing when. And why different representational commitments succeed or fail. In doing so, it offers a step toward a more integrated science of reasoning that can accommodate contextual dependence without sacrificing explanatory precision.

## Data Availability

The original contributions presented in the study are included in the article/supplementary material, further inquiries can be directed to the corresponding author.
